# Genotype–Phenotype Correlations, Treatment, and Prognosis of Children With Early‐Onset (Neonatal) Marfan Syndrome

**DOI:** 10.1111/cge.14722

**Published:** 2025-03-10

**Authors:** Eva C. van der Leest, Annelies E. van der Hulst, Gerard Pals, Lidiia Zhytnik, Lillian Lai, Caroline Jacquemart, Lindsay Mills, Michiel Houben, Petr Jira, Bert L. Lunshof, Jessica Warnink‐Kavelaars, Vivian de Waard, Leonie A. Menke

**Affiliations:** ^1^ Department of Pediatrics Amsterdam UMC, University of Amsterdam, Emma Children's Hospital Amsterdam the Netherlands; ^2^ Amsterdam Reproduction & Development Amsterdam the Netherlands; ^3^ Department of Pediatric Cardiology Amsterdam UMC, University of Amsterdam, Emma Children's Hospital Amsterdam the Netherlands; ^4^ Department of Human Genetics Amsterdam UMC, Vrije Universiteit Amsterdam Amsterdam the Netherlands; ^5^ Rare Bone Disease Center Amsterdam Amsterdam the Netherlands; ^6^ Amsterdam Movement Sciences, Rehabilitation and Development Amsterdam the Netherlands; ^7^ Department of Endocrinology and Metabolism Amsterdam UMC, Vrije Universiteit Amsterdam Amsterdam the Netherlands; ^8^ Department of Traumatology and Orthopaedics The University of Tartu Tartu Estonia; ^9^ Division of Cardiology Children's Hospital of Eastern Ontario, University of Ottawa Ottawa Ontario Canada; ^10^ Department of Pediatrics Centre Hospitalier Universitaire de Liège, Pediatric Cardiology Liège Belgium; ^11^ Division of Cardiology, Department of Pediatrics University of Calgary Calgary Alberta Canada; ^12^ Department of Pediatrics University Medical Center, Wilhelmina Kinderziekenhuis Utrecht the Netherlands; ^13^ Department of Pediatrics Jeroen Bosch Ziekenhuis 's‐Hertogenbosch the Netherlands; ^14^ Department of Pediatrics Gelre Ziekenhuizen Apeldoorn the Netherlands; ^15^ Department of Rehabilitation Medicine Amsterdam UMC, University of Amsterdam Amsterdam the Netherlands; ^16^ Department of Medical Biochemistry Amsterdam UMC, University of Amsterdam Amsterdam the Netherlands; ^17^ Amsterdam Cardiovascular Sciences Atherosclerosis and Ischemic Syndromes Amsterdam the Netherlands; ^18^ Amsterdam Neuroscience—Cellular & Molecular Mechanisms Amsterdam the Netherlands; ^19^ Emma Center for Personalized Medicine Amsterdam the Netherlands

**Keywords:** cardiac valve annuloplasty, Fibrillin‐1, genotype–phenotype correlations, heart defects congenital, Marfan syndrome, mitral valve insufficiency, prognosis, tricuspid valve insufficiency

## Abstract

Early‐onset Marfan syndrome (eoMFS) is a severe and rare form of Marfan syndrome characterized by severe atrioventricular valve insufficiency developing before or shortly after birth. It is unclear which factors (interventions and/or genotype) influence survival. Forty‐one individuals with eoMFS with a fibrillin‐1 gene (*FBN1*) variant in exon 24–32 (CRCh37) were included. At the last follow‐up, 14/41 (34%) were alive (8 months‐18 years) and 27/41 (66%) were deceased. Median age of death was 1 month and 88% of the deaths occurred before 5 months of age. More individuals alive past the age of 16 months versus those who were deceased before that age had undergone cardiovascular surgery at an older age (13 months, range 3–72, vs. 2 months, range 2–2, *p* = 0.03). Survival was better in those with single amino acid substitutions/small in‐frame deletions than in those with large in‐frame deletions (*p* = 0.007), but variants involving a cysteine substitution in an EGF‐like domain versus those involving other amino acids did not significantly influence survival. EoMFS ranges from a (pre‐)neonatal life‐threatening disorder to a disorder with enhanced survival, creating a window for cardiovascular surgery. Individuals with single amino acid substitutions/small in‐frame deletions had better survival compared to those with variants significantly impacting exon 24–32 length.

## Graphical abstract

Early‐onset Marfan syndrome (eoMFS) is a rare disorder with atrioventricular valve insufficiency being the most severe symptom. We propose to regard eoMFS as a spectrum, ranging from a severe disorder life‐threatening already before or immediately after birth, to a disorder with a better survival rate, creating a window for atrioventricular valve surgery.

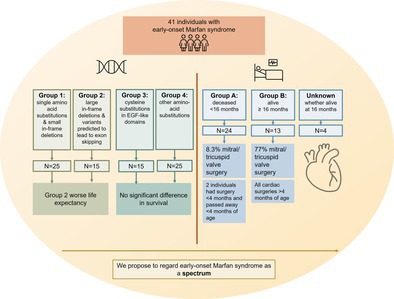

## 1 Introduction

Marfan syndrome (MFS) is a connective tissue disorder caused by variants in fibrillin‐1 (*FBN1*) characterized by ocular and skeletal manifestations and aortic root (AoR) dilation with a high risk of dissections before the age of 40 if left untreated [1]. Early‐onset MFS (eoMFS, also known as neonatal MFS or infantile MFS), a severe and rare form of MFS, is characterized by prolapse of the atrioventricular valves (AV‐valves), resulting in significant AV‐valve insufficiency (AVI) with or without AoR dilatation, before or shortly after birth [2, 3]. Like classical MFS, it is caused by pathogenic variants in the *FBN1* gene. An overrepresentation of *FBN1* variants causing eoMFS is found to be located in exons 24–32 [4]. However, not all variants in exons 24–32 of *FBN1* lead to eoMFS, since many *FBN1* variants in these exons are also reported for classical MFS [5] without prominent AVI.

To diagnose eoMFS rather the presence of mitral (MV) or tricuspid valve (TV) insufficiency is an important determinant [1, 2, 6, 7, 8, 9]. Until recently, there was no consensus on the age of onset or severity of AVI to qualify for a diagnosis of eoMFS [3, 4, 6, 10, 11]. Zarate et al. recently proposed diagnostic criteria for eoMFS [12] in which severe MVI and/or TVI before 1 year of age gives a higher score fitting a greater probability of eoMFS.

Thirty years ago, a mean age of death of 16.3 months (range 0–14 years) was calculated based on 86 published individuals with eoMFS [7]. Comparable results were shown in a recent review of 54 children with eoMFS [13]. Forty‐seven (87%) children passed away before 16 months of age, and only five were still alive (2, 8, 9, 10 and 14 years old). Additional case reports described children still alive at 4 years (*n* = 2) and 11 years of age [11, 14, 15]. Since heart failure as a result of AVI is the major cause of death in eoMFS patients [1], we hypothesized that survival might depend on surgical intervention once mild to moderate AVI is detected, instead of waiting until AVI progresses. Alternatively, survival may depend on genotype–phenotype correlations within the eoMFS group. For example, variants leading to cysteine substitution in EGF‐like domains are associated with disease severity in individuals with MFS [16].

Apart from studying survival in eoMFS in relation to cardiovascular interventions and genotype, we aimed at describing the non‐cardiovascular characteristics to identify additional symptoms while growing older and to identify factors that might be associated with a better prognosis. We gathered extensive data from nine eoMFS individuals (of whom two were previously reported [17, 18]) and reviewed the literature to identify 32 additional patients with eoMFS eligible for our extensive analyses.

## 2 Methods

### 2.1 Patients

Individuals were included if (1) the child had a pathogenic or likely pathogenic variant in exon 24–32 of *FBN1* (GRCh37), and (2) had developed significant MV insufficiency (MVI) and/or TV insufficiency (TVI) before 1 year of age, and (3) information on the grade of AVI and/or AoR diameter was available. Eligible individuals were found via the national and international academic network of the researchers, and via Genesis, a registry for genetic diagnoses used at the Amsterdam UMC expertise center for MFS and related disorders. The registry was consulted for individuals with a variant in exon 24–32 of *FBN1* in whom genetic testing had been requested before 1 year of age to prevent finding too many individuals with MFS rather than eoMFS.

A Pubmed search was performed using the following keywords: “neonatal Marfan”, “infantile Marfan”, “congenital Marfan”, “early‐onset Marfan”, “early‐onset Marfan”, “mitral insufficiency” and “Marfan”, “mitral regurgitation” and “Marfan”, “tricuspid insufficiency” and “Marfan”, and “tricuspid regurgitation” and “Marfan”. The title and abstract of the papers were screened, and if unclear whether an individual met the inclusion criteria, the full text was screened, by one author (ECL). All children whose data was retrieved from the literature only, received a patient number preceded by the letter “L” to distinguish them from patients from whom we received more extensive data through their health care professionals. The latter individuals are represented by a patient number only.

Data was gathered and scored retrospectively using a patient report form that was filled out by the pediatric cardiologist or pediatrician. For individuals known in the Amsterdam UMC and individuals from literature, data was gathered by one author (ECL).

### 2.2 Phenotype

Collected patient information included demographics, genetic variant, age at diagnosis, symptoms, the grade of AVI and AoR diameter at different time points, interventions (medication, cardiovascular and non‐cardiovascular interventions), and current age or age at death and cause of death. Ultrasound information was gathered for different time points (if available) until the last consultation. Time points included, but were not limited to: neonatal age, moment of diagnosis, before surgery (if applicable), and at last consultation. Other characteristics, symptoms, and interventions were only recorded if present until the last consultation. Echocardiographic data (AoR diameter, AVI grade, etc.) from individuals known at Amsterdam UMC were scored by one pediatric cardiologist (AEH) and those from other individuals were scored by the local pediatric cardiologists. Detroit reference values [19] were used for the calculation of Z‐scores of the aortic valve annulus, AoR (sinus of Valsalva), and sino‐tubular junction (ST‐junction). Halifax reference values [20] were used for the calculation of Z‐scores of the ascending aorta. AVI grade was interpreted by the local pediatric cardiologist: grade 1 “mild insufficiency”, grade 2 “moderate insufficiency”, grade 3 “severe insufficiency, grade 4 ‘very severe insufficiency’” [21].

We scored all nine enrolled individuals with the novel clinical scoring system for eoMFS (a score > 16 points being indicative of eoMFS) [12] for conformity reasons. Data was collected using Castor EDC [22].

### 2.3 Genotype

Alamut Visual, version 2.15 (SophiaGenetics.com) was used to create an overview of the genetic variants at DNA and protein level, based on transcript NM_000138.4, according to the HGVS guidelines (https//www.hgvs.org). In the more recent genomic reference sequences of *FBN1* the numbering of exons is shifted, since the non‐coding exon number one has been added. To prevent confusion with existing literature, we used the old numbering, with exon 24–32 (rather than 25–33) representing the neonatal region in *FBN1*. Yin‐O‐Yang software versus 1.2 (DTU Health Tech: https://services.healthtech.dtu.dk/service.php?YinOYang‐1.2) was used to predict the effect of variants involving serine or threonine on O‐glycosylation, which is predicted to have a relatively large impact on the 3D‐structure of the protein.

The pathogenic effect of the *FBN1* variants was assessed according to the ACMG/AMP *FBN1* VCEP variant classification [23] for conformity reasons. However, all variants were also assessed according to the consensus among MFS experts, as explained in Franken et al. [24]. In short, all exons in the region exon 7 through 63 are multiples of 3 nucleotides, so exon‐skipping mutations do not lead to frameshift and nonsense‐mediated decay (NMD). The result is a truncated protein, which is a known mechanism of pathogenic variants in *FBN1*. Changes in the consensus sequence of any of the 44 calcium‐binding EGF‐like domains are also a known mechanism of pathogenic variants [25]. Evidence of exon skipping was determined on cDNA from cultured fibroblasts. For most splice‐site variants, no mRNA analysis data was available. We assumed that variants in the canonical splice‐sites involving the +1GT start or the −2AG end of an intron would always lead to a large in‐frame effect on the mRNA, as variants in the canonical splice‐sites always affect splicing [26]. Exon skipping in the neonatal region of *FBN1* always results in an in‐frame deletion with a severe effect [24]. Use of a cryptic splice‐site may result in a large in‐frame deletion or insertion [24]. Genetic variants were visualized in Figure 3, created using Lucidchart (Lucidchart.com).

### 2.4 Statistical Analysis

All individuals were divided into two groups: those who deceased before 16 months (A) and those reported alive at 16 months of age (B), to identify factors associated with survival past this age. We chose 16 months as the cut‐off age, according to the mean age of death in the largest cohort of children with eoMFS [7]. Fisher exact tests were used to compare frequencies and Mann–Whitney *U* tests to compare medians. Independent‐samples median tests were used to compare AVI grades.

To investigate genotype–phenotype correlations, variants were divided into four (overlapping) groups: (1) single amino acid substitutions/small in‐frame deletions, (2) exon‐skipping and large in‐frame deletions or insertions, (3) substitutions involving a cysteine in an EGF‐like domain, and (4) substitutions involving other amino acids. Groups 3 and 4 did not include splice‐site variants. Kaplan–Meier curves were constructed to visualize the survival in each of these groups. The Log‐rank test was used to test whether outcomes were significantly different between group 1 versus 2, and group 3 versus 4. P‐values < 0.05 were considered statistically significant. IBM SPSS Statistics (version 26 for iOS) was used for all analyses [27].

## 3 Results

### 3.1 Patients

We identified 41 individuals with eoMFS. First, we gathered information on nine individuals with eoMFS of whom two individuals turned out to have been described in literature before [17, 18]. All nine individuals had a score ≥ 16 points indicating a diagnosis of eoMFS according to the novel scoring system of Zarate et al. [12] (Table S1). A complete description of these individuals is provided in Table S2. Second, we identified 32 additional individuals from literature. Figure S2 shows a flow chart of the literature search and reasons for in‐ and exclusion. Table S3 provides the references for these 32 individuals. Table S5 reports the main clinical manifestations and surgical interventions performed in the 41 individuals.

**FIGURE 1:**
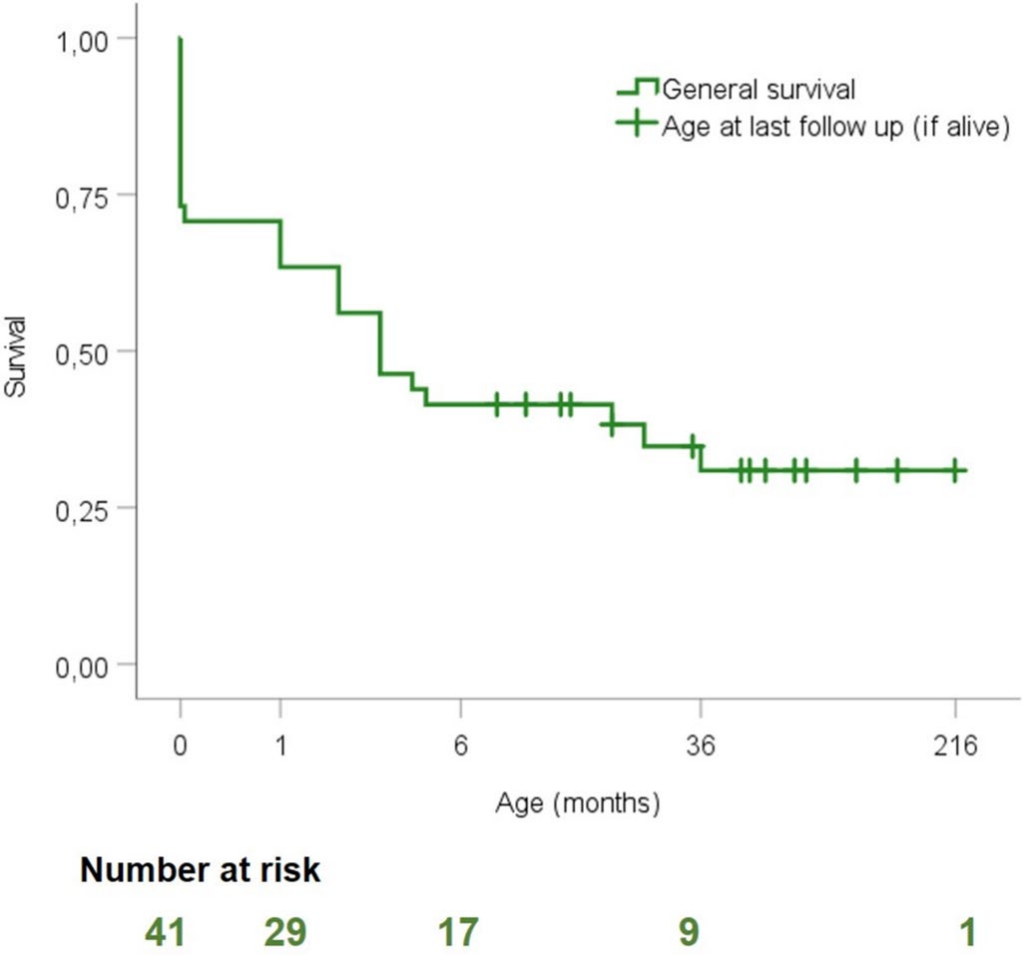
Kaplan–Meier survival curve of the presently described children with early‐onset Marfan syndrome. *X*‐axis represents survival in months and *Y*‐axis represents cumulative survival.

At the last follow‐up, fourteen (34%) of the 41 individuals were alive with a median age of 4 years (range 8 months‐18 years). Twenty‐seven (66%) of the 41 individuals were deceased with a median age of death of 1 month (range 0–36). The first cardiac ultrasound (made within 6 months after birth) showed a median AoR Z‐score of +4.9 (*n* = 8) and a median AVI grade of 2 (*n* = 26). Figure 1 shows the Kaplan–Meier survival curve depicting a rapid decrease in cumulative survival within the first months of life with 88% [22/27] of the deaths occurring before 5 months of age, mostly of cardiac and/or respiratory failure due to AVI. Figure S3 shows survival over the last 30 years. Most individuals who were deceased < 16 months were born more than 10 years ago, before 2014 [88%, 21/24], while most individuals alive ≥ 16 months were born after 2014, or are cases from studies published after 2014 [85%, 11/13].

### 3.2 Phenotype: Cardiovascular Medication and Surgical Interventions

Table 1 shows the clinical characteristics and cardiovascular interventions of the children deceased prior to 16 months of age (group A, *n* = 24) compared to those alive beyond that age (group B, *n* = 13). Note that 4/41 individuals were not included in group A or B, since they were younger than 16 months at the last follow‐up and still alive. The individuals from group A were diagnosed with eoMFS at a younger age than those from group B [median 0 vs. 1 month; *p* = 0.003]. Insufficient data was available to compare AVI grades or AoR dilatation between groups A and B.

**TABLE 1:** Characteristics of the presently described cohort of children with early‐onset Marfan syndrome.

	Total	Deceased < 16 months	Alive at 16 months	
	*n* = 41	*n* = 24	*n* = 13^K^	*p*
Age at diagnosis (months)^A^	0 [IQR = 1, *n* = 35]	0 [IQR = 0, *n* = 21]	1 [IQR = 7, *n* = 11]	0.003 [MW]
Gender (female)	35% [13/37]	38% [8/21]	23% [3/12]	0.687 [F]
Deceased^B^	66% [27/41]	100% [24/24]	23% [3/13]	0.001 [F]
Age at moment of death (months)^C^	1.00 [IQR = 3.0, *n* = 25]	1.0 [IQR = 3.0, *n* = 22]	24.0 (IQR = N/A, *n* = 3)	0.001 [MW]
**Clinical features at birth**				
Pectus deformity^D^	34% [14/41]	21% [5/24]	54% [7/13]	0.067 [F]
Congenital flexion contractures	63% [26/41]	79% [19/24]	31% [4/13]	0.006 [F]
Ear deformities^E^	56% [23/41]	71% [17/24]	38% [5/13]	0.083 [F]
Loose and/or redundant skin	63% [26/41]	67% [16/24]	62% [8/13]	1.000 [F]
Long fingers	98% [40/41]	100% [24/24]	92% [12/13]	0.351 [F]
**Other clinical characteristics**				
Myopia	92% [11/12]	50% [1//2]	100% [10/10]	0.167 [F]
Ectopia Lentis	48% [13/27]	29% [4/14]	72% [8/11]	0.047 [F]
Pneumothorax	30% [7/23]	29% [4/14]	38% [3/8]	1.000 [F]
Lung emphysema^F^	48% [11/23]	57% [8/14]	38% [3/8]	0.659 [F]
Diaphragmatic hernia/ eventration	33% [7/21]	33% [4/12]	43% [3/7]	1.000 [F]
Scoliosis	74% [14/19]	33% [2/6]	91% [10/11]	0.028 [F]
Pes planovalgus	80% [12/15]	67% [4/6]	86% [6/7]	0.559 [F]
**Development**				
Mean age at first words (months)	12 (IQR = 7, *n* = 6)	N/A	12 (IQR = 7, *n* = 6)	N/A
Mean age at first walking (months)	19 (IQR = 15, *n* = 6)	N/A	19 (IQR = 15, *n* = 6)	N/A
**Cardiac drugs**	66% [27/41]	33% [8/24]	100% [13/13]	0.001 [F]
β‐blocker	44% [18/41]	17% [4/24]	92% [12/13]	0.001 [F]
ARB	34% [14/41]	8% [2/24]	85% [11/13]	0.001 [F]
ACE inhibitor	17% [7/41]	13% [3/24]	7% [1/13]	1.000 [F]
Age start cardiac drugs (months)	4.0 (IQR = 9.0, *n* = 23)	0.0 (IQR = 2.0, *n* = 9)	8.0 (IQR = 8.0, *n* = 11)	0.001 [MW]
**Echocardiography at diagnosis** ^G^				
Mitral insufficiency (grade)	2.0 (IQR = 2.0, *n* = 27)	2.0 (IQR = 2.5, *n* = 17)	1.0 (IQR = 2.0, *n* = 9)	0.357 [IM]
Tricuspid insufficiency (grade)	2.0 (IQR = 2.0, *n* = 26)	2.0 (IQR = 3.0, *n* = 17)	1.0 (IQR = 1.8, *n* = 8)	0.362 [IM]
Aortic root dilatation (Z‐score)	4.89 (IQR = 2.6, *n* = 8)	5.49 (IQR = N/A, *n* = 2)	5.18 (IQR = 2.6, *n* = 5)	1.000 [MW]
**Echocardiography before cardiac surgery** ^H^				
Mitral insufficiency (grade)	3.5 (IQR = 2.3, *n* = 10)	3.0 (IQR = N/A, *n* = 2)	3.0 (IQR = 3.0, *n* = 7)	1.000 [IM]
Tricuspid insufficiency (grade)	3.0 (IQR = 2.3, *n* = 8)	3.5 (IQR = N/A, *n* = 2)	3.0 (IQR = 2.0, *n* = 5)	0.286 [IM]
Aortic root dilatation (Z‐score)	5.31 (IQR = 3.0, *n* = 5)	N/A	6.16 (IQR = 2.5, *n* = 4)	N/A
**Cardiac surgery**	37% [15/41]	8.3% [2/24]	77% [10/13]^L^	0.001 [F]
Mean age at first cardiac surgery (months)^I^	10 (IQR = 10, *n* = 15)	2.0 (IQR = N/A, *n* = 2)	13 (IQR = 48, *n* = 10)	0.030 [MW]
**Other surgeries**				
Inguinal hernia repair	16% [6/38]	4.2% [1/24]	33% [4/12]	0.034 [F]
Scoliosis surgery	13% [5/38]	0% [0/24]	42% [5/12]	0.002 [F]
Pyloromyotomy	7.9% [3/38]	8.3% [2/24]	8.3% [1/12]	1.000 [F]
Diaphragm plication	5.3% [2/38]	4.2% [1/24]	8.3% [1/12]	1.000 [F]
Ocular surgery^J^	7.9% [3/38]	0% [0/24]	25% [3/12]	0.031 [F]
**Other therapies**				
Tube feeding	40% [8/20]	18% [2/11]	63% [5/8]	0.074 [F]
Thoracic casting/corset/brace	16% [5/32]	0% [0/21]	50% [5/10]	0.001 [F]
Ankle/ft orthotics	16% [5/32]	0% [0/21]	50% [5/10]	0.001 [F]
Physiotherapy	13% [4/32]	0% [0/21]	40% [4/10]	0.007 [F]
Thumb brace	3.1% [1/32]	0% [0/21]	10% [1/10]	0.323 [F]
(Semi) orthopedic shoes	6.3% [2/32]	0% [0/21]	20% [2/10]	0.097 [F]

**Abbreviations:** ACE‐inhibitor, Angiotensin‐converting‐enzyme inhibitor; ARB, Angiotensin II receptor blocker; Cardiac surgery, AV‐valve and/or AoR surgery; F, Fisher's Exact Test; IM, Independent‐Samples Median Test; IQR, Interquartile range; MW, Mann‐Whitney U test; N/A, Not available/Not applicable; **Superscripts** A, Age at diagnosis in months. Twice prenatal diagnosis, not in results; B&C, Age at moment at death in months. One labor induction 26 weeks, not in results; D, Pectus deformity, pectus excavating deformity of the chest, or pectus carinatum; E, Ear deformity, e.g. long and not firm and over folded helix, crumpled ears with much cartilage, smooth and flat ears, or abnormal auricles; F, Lung emphysema is not always proven, but sometimes predicted because of recurrent respiratory infections; G, Including echocardiography made up to age 5 months, in case children were diagnosed later; H, Only data from indiviuals who had cardiac surgery. Evaluation was performed up to one month before (first) cardiac surgery; I, Age in months at the moment of first AV‐valve surgery or AoR surgery; J, Ocular surgery, one lens removal, or bilateral cataract extraction surgery; K, Excluding 4 individuals who were still alive but had not yet reached the age of 16 months; L, One of the individuals who did not have cardiac surgery, was deceased at last follow up.

**FIGURE 2:**
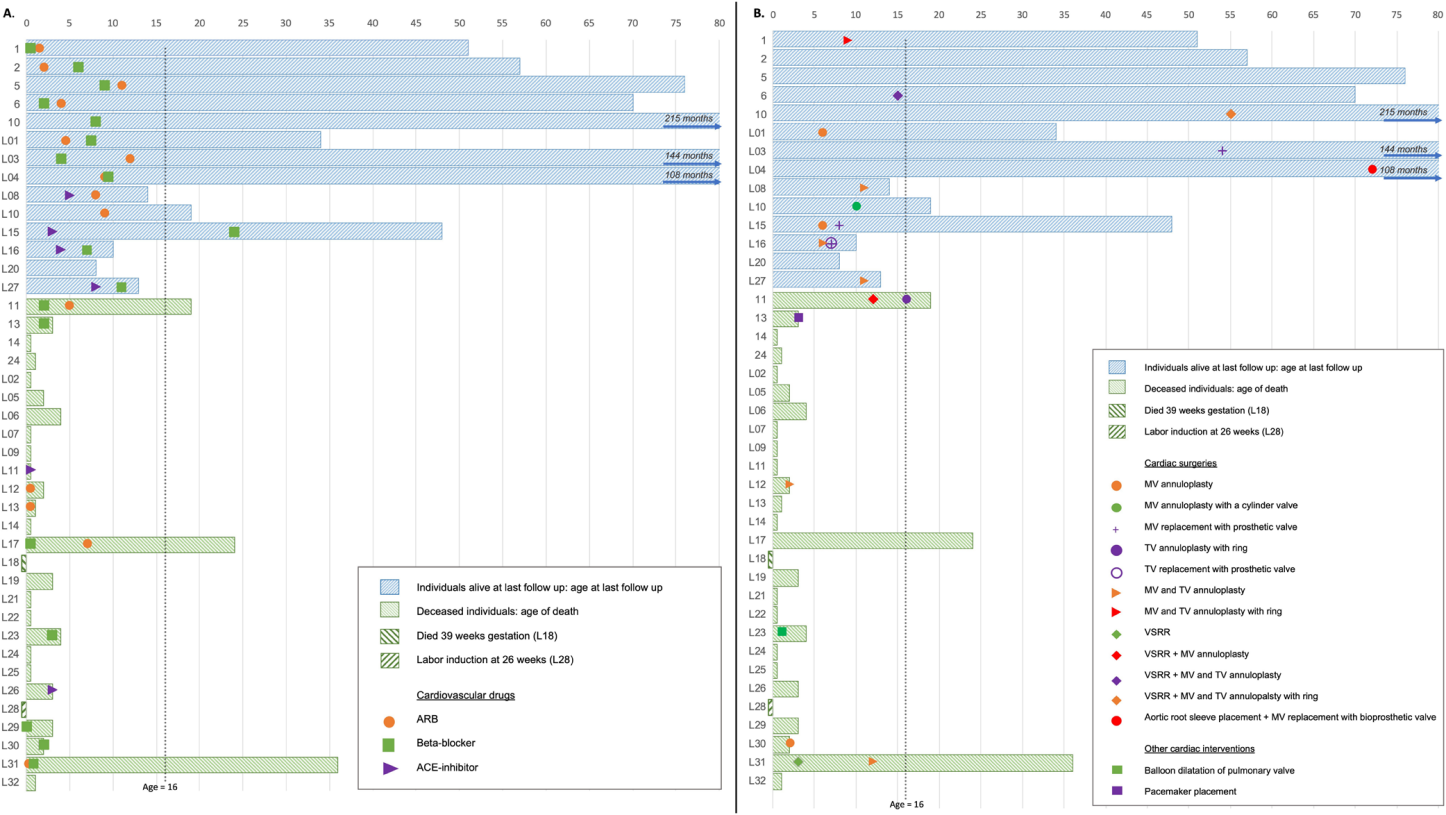
Diagram showing age in months at which cardiac interventions were commenced per individual. A. Types of cardiac drugs and B. Types of cardiac surgeries. Dotted line represents 16 months of age. Individual 10, L03 and L04 were alive at 100 months of age. Age at the last follow‐up is marked with an arrow. A few individuals from literature (L22, L24, L25, L26) had medical treatment for congestive heart failure or respiratory failure, which were not specified and therefore not shown in this study. Individual L10 started before age 9 months with an ARB, specific moment unknown.

Figure 2A shows the prescription of β‐blockers, Angiotensin II Receptor type 1 Blockers (ARB), and Angiotensin‐Converting‐Enzyme (ACE) inhibitors in all children (*n* = 41). Prophylactic cardiovascular medication was prescribed more often in group B versus group A [100% vs. 33%; *p* = 0.000] and the age of onset of treatment was higher in group B versus group A [8 months vs. 0 months, *p* = 0.001]. Almost all children in group B received a β‐blocker, an ARB, or both. Fourteen children received diuretics [34%; 14/41] or inotropic drugs [37%; 15/41], mostly for (acute) congestive heart failure or for a short period of time to reduce cardiac afterload before and after cardiac surgery.

Figure 2B shows the type of cardiovascular surgical procedures performed in all children. Considering all 41 eoMFS individuals, 37% underwent AV‐valve annuloplasty and/or replacement [15/41], of which 11 individuals were alive at the last follow‐up. Among the individuals who underwent AV‐valve surgery, a third [5/15] underwent AoR surgery as well. In six individuals, the MV but not the TV had been operated on; the other nine individuals underwent surgery on both AV‐valves. One child did not receive any AV‐valve surgery, but had a pacemaker implanted after an event of sustained asystole. She passed away shortly after this intervention as a result of cardiac failure (individual 13). More individuals from group B underwent cardiac surgery [77% vs. 8%; *p* = 0.001] at a relatively older age [13, range 3–72, vs. 2, range 2–2, months; *p* = 0.030] compared to those from group A.

### 3.3 Phenotype: Non‐Cardiovascular Characteristics and Interventions

Table 1, Figure S1, and Table S5 show the prominent morphological features at birth. Congenital joint contractures were present more often in group A versus group B [79% vs. 31%, *p* = 0.006]. During development, individuals faced various challenges apart from their cardiovascular problems such as feeding problems, delayed motor development, ectopia lentis, lung emphysema, and scoliosis. In general, group B received more interventions than group A, including thoracic casting and/or spinal surgery, inguinal hernia repair, and ocular surgery (Tables 1 and S5). The eldest individual (currently 18 years old) required spinal surgery five times.

### 3.4 Genotype

**FIGURE 3:**
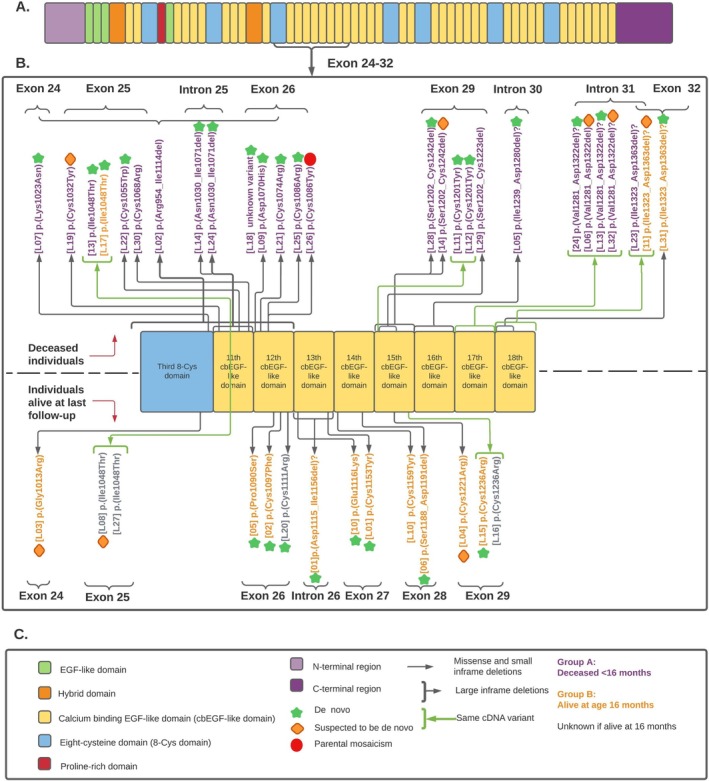
Schematic presentation of the FBN1 protein domain structure, distribution of the genetic variants, and exons/introns encoding the domains. (A) The FBN1 protein and its domains. (B) The variant effect on the protein with IDs of the individuals between brackets. The exon‐skipping effects are confirmed, except for those marked with a question‐mark; those are predicted. Above dotted line; individuals deceased at the last follow‐up, and below the dotted line; individuals alive at the last follow‐up. The exons/introns where the corresponding encoding cDNA variants are located, are shown in horizontal text in higher font size. If known there is indicated whether a variant is de novo (mutation was not found in either of the parents) or suspected to be de novo (parents were clinically unaffected, but not screened). (C) Symbol legend.

**FIGURE 4:**
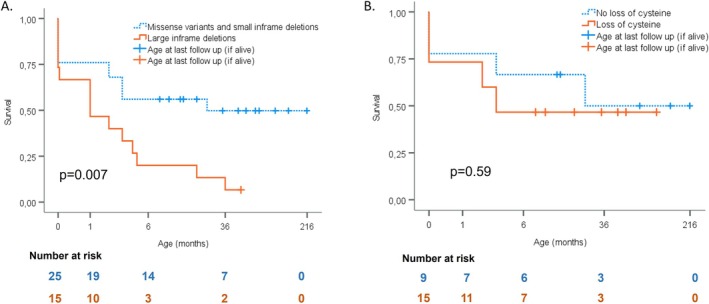
Kaplan–Meier survival curves for different *FBN1* variants. *X*‐axis represents survival in months and *Y*‐axis represents cumulative survival. (A) Individuals with missense variants or small in‐frame deletions (dotted blue) versus individuals with large in‐frame deletions or variants predicted to lead to exon skipping (orange). (B) Individuals with missense variants not leading to a loss of a cysteine (dotted blue) versus individuals with loss‐of‐cysteine missense variants (orange). For L18, only a ‘de novo genetic variant in exon 26’ was reported. Therefore, this individual could not be included in the survival analysis.

The *FBN1* variants, subdivided by survival outcome, are shown in Figure 3 (extended data in Table S6). *FBN1* variants were either *de novo*, suspected *de novo* (parents did not show signs of MFS), or unknown. Most variants were single amino acid substitutions or small in‐frame deletions [25/40, 63%] (group 1), and other variants were large in‐frame deletions or variants predicted to lead to exon skipping [15/40, 38%] (group 2). Twenty‐three individuals were born before 2014 (Figure S3), of whom ten were from group 2. Survival analysis showed that group 2 had a worse life expectancy than group 1 (Figure 4A). When comparing all missense variants involving a cysteine substitution in an EGF‐like domain (only loss‐of‐cysteine variants were found, no gain‐of‐cysteine variants) [15/40, 38%] (group 3) to those involving other amino acids [9/40, 23%] (group 4), survival was not significantly different (Figure 4B).

Most variants were in exon 25 (*n* = 7), exon 26 (*n* = 7) and in intron 31 (*n* = 7). One large genomic deletion also included these exons (deletion of exon 24–26). Another large deletion extended from intron 31 into exon 32, resulting in the removal of the splice‐acceptor‐site of exon 32, and leading to the loss of exon 32. Variants specifically leading to loss of exon 31 or 32 (*n* = 7) were all found in the group deceased before 16 months. Individuals with variants in exon 27 (*n* = 2) or 28 (*n* = 2) all survived the first 16 months of life.

The clinical outcome of five recurrent variants (Figure 3) is described in Table S4.

## 4 Discussion

We collected data of 41 children with eoMFS with a genetic variant in the neonatal region (exon 24–32) of the *FBN1* gene and significant AVI before 1 year of age. Sixty‐six percent were deceased due to cardiac and/or respiratory failure. Most children were deceased in the first 5 months of life, while a number of children survived past the first 16 months of life, indicating a wide disease spectrum. Children with a less severe outcome started using cardiac drugs and/or underwent cardiac surgery at an age at which children in the group with a worse outcome were already deceased. Variants of the deceased children were located in regions exon 24–26 and exon 29–32, and individuals with single amino acid substitutions/small in‐frame deletions had better survival compared to those with variants significantly impacting exon 24–32 length. The children who outlived the first months of life also developed various non‐cardiovascular health issues requiring frequent medical attention.

Survival in the 41 children with eoMFS varied from 0 days to alive at almost 18 years, but most children [88%, 22/27] died within the first 5 months as a result of heart failure due to severe AVI. However, the outcome for children who survived past this age is much better than previously thought [7]. Our study shows that surviving the first year of life seems to greatly increase the life expectancy of children with eoMFS. We hypothesize that cardiomyocyte proliferation and maturation may play a role in this discrepancy. The cardiac tissue and AV‐valves in all children with eoMFS are very fragile [18]. In healthy infants, cardiomyocyte proliferation, and reorganization of the cytoskeleton and extracellular matrix (ECM) are important to absorb the increase in systemic afterload after birth [28]. The proliferation capacity of cardiomyocytes sharply declines after the first three months of life, to almost nonexistent after 1 year [29]. Fibrillin‐1 fibers form an essential part of the cardiac ECM and control transforming growth factor‐mediated signaling [30, 31], a key pathway during cardiac (valve) development and maturation [32]. The cardiac tissue of children with a milder form of eoMFS may still have the ability to adapt and regenerate to some extent, while in the most severely affected children, it is too greatly impaired. Novel fundamental research is needed to investigate this hypothesis.

Our study showed that all children alive at 16 months received an ARB or β‐blocker, mostly prescribed as prophylactic therapy to reduce AoR growth. Lacro et al. found an AoR Z‐score lowering effect for both β‐blocker and ARB as a single treatment in a large group of children with *classical* MFS [33]. They also showed a greater decrease in the AoR Z‐score over time for both drugs, when started at a younger age. The positive effect on the AoR Z‐score of the combination of an ARB and a β‐blocker in a child with eoMFS has also been shown [34]. Since we had limited data available and most children received medication from diagnosis, we cannot conclude if the medication reduced AoR growth.

Seventy‐seven percent of the children alive at 16 months had undergone MV and/or TV surgery, versus 8.3% of the children who deceased before 16 months. Furthermore, our results showed that most individuals deceased < 16 months were born before 2014 [88%, 21/24], and most individuals alive ≥ 16 months were born after 2014 [85%, 11/13]. Different types of genetic variants were evenly distributed and could not have caused this difference in survival. So, this improvement in survival in more recent years may reflect the improvements in medical care. However, genetics likely also plays a role, since even in recent years some neonates deceased directly or shortly after birth (L24, L28, L32). Also, four individuals with the same genetic variant, all born in a different decade (24, L06, L13, L32), were deceased before 4 months of age.

With regard to intervention, all AV‐valve surgeries were performed after the age of 4 months, except for two individuals. Yet, these two children did pass away before 4 months of age. This may indicate that some children are too severely affected to survive, despite cardiac surgery. In a newborn with eoMFS, we currently cannot predict reliably how severe the prognosis will be, who will best benefit from surgery, and at which time point surgical intervention should be performed. Since surgical intervention is strongly associated with survival in eoMFS children, it should be considered to prevent advanced cardiac damage.

The children with eoMFS who survived the first few months faced a variety of physical problems. Hypermobility and frequent hospitalizations probably accounted for the delayed motor development. Tube feeding was often required (~50%). In half of the individuals, lung emphysema was suspected, which has not been consistently reported in the literature [6, 35]. In half of the children, ectopia lentis was found, which is similar to classical MFS due to *FBN1* variants in exon 24–32 (53%) [9]. Scoliosis was present in 74% of the children, much more than reported for classical MFS due to *FBN1* variants in exon 24–32 (38%) [36]. Congenital joint contractures were present more often in the most severe eoMFS children. Whether this feature could be used as an indicator of severity, should be investigated in future studies.

Only 38% of the *FBN1* variants led to a loss of an essential cysteine in an EGF‐like domain, yet this did *not* affect survival. This was not expected, since in a recent study—describing genotype–phenotype correlations of 1557 individuals with classical MFS—cysteine substitutions in the neonatal region contributed 54% and aortic/MV surgery was performed more and at an earlier age in individuals with variants leading to cysteine substitutions [16]. Elsäcker et al. [37] studied AoR dimensions in 97 children with MFS (age 0–20 years) and found that AoR size was larger in individuals with MFS with *FBN1* variants resulting in a cysteine loss, than with variants resulting in a gain of cysteine or not affecting a cysteine. This was similar in adults with classical MFS [16]. Interestingly, among the eoMFS *FBN1* variants in our study, there were no gain‐of‐cysteine *FBN1* variants.

Contrarily, we found 15 children (38%) with large in‐frame deletions or splice‐site variants (predicted to lead to exon skipping), having a worse life expectancy than children with other variants in the neonatal region. Together with the cysteine substitutions, these in‐frame *FBN1* variants are considered dominant‐negative (DN; altered fibrillin‐1 protein structure). In a French [16] and Dutch [24] *classical* MFS cohort of 1575 and 570 individuals respectively, 41% of the *FBN1* variants were predicted to cause haploinsufficiency (HI; reduced fibrillin‐1 protein levels) and a more severe aortic phenotype [16, 24]. For eoMFS, *FBN1* variants with a predicted DN phenotype cause the very severe cardiac phenotype, since no HI mutations were found within the eoMFS group. Similarly, Faivre et al. investigated genotype–phenotype correlations in 1013 individuals, including 43 individuals with eoMFS, and described that HI *FBN1* variants are rarely associated with eoMFS [9]. Since it is easier to predict which mutations will result in HI, as compared to the effect of the highly variable DN mutations on protein function, it becomes clear that DN *FBN1* variants can cause a wide range of cardiovascular pathology, from eoMFS to relatively mild classical MFS in adults. The recent review by Chen et al. on genotype–phenotype correlations substantiates what we describe [38]. We have summarized our genotype–phenotype findings in Table 2, comparing them to two large classical MFS studies.

**TABLE 2:** Summary of our genetic findings compared to two large classical Marfan syndrome studies.

	Early‐onset Marfan syndrome	Classical Marfan syndrome children	Classical Marfan syndrome adults
	This study	Van Elsäcker et al. [36]	Arnaud et al. [16]
Cohort	*N* = 41	*N* = 97	*N* = 1575
Most severe cardiovascular phenotype	Cardiac	Aorta	Aorta
Survival	34% at last follow‐up	No deaths	90% at 60 years
Genotype	Exon 24–32^A^	Whole *FBN1* gene	Whole *FBN1* gene
	Only dominant‐negative in‐frame *FBN1* variants	55% dominant‐negative predicted *FBN1* variants	59% dominant‐negative predicted *FBN1* variants
	No haploinsufficient *FBN1* variants	37% haploinsufficient predicted *FBN1* variants	41% haploinsufficient predicted *FBN1* variants
	No gain‐of‐cysteine *FBN1* variants	Both loss‐of‐cysteine and gain‐of‐cysteine *FBN1* variants	Both loss‐of‐cysteine and gain‐of‐cysteine *FBN1* variants
Genotype–Phenotype	Large in‐frame insertions/ deletions in exon 24–32 region associate with decreased survival due to cardiac and/or respiratory failure caused by atrioventricular valve insufficiency	No difference in aortic disease severity between dominant‐negative and haploinsufficient *FBN1* variants	Haploinsufficient *FBN1* variants associate with decreased survival due to aortic events as compared to dominant‐negative *FBN1* variants
	Loss‐of‐cysteine variants do not impact disease severity more than other amino acid substitutions	Loss‐of‐cysteine variants lead to more severe aortic phenotype as compared to gain of cysteine or other amino acid substitutions	Loss‐of‐cysteine variants lead to more severe cardiac and aortic phenotype as compared to gain of cysteine or other amino acid substitutions

*Note*: A: Phenotype is not solely caused by variants in this region. However, in this study only individuals with variants in this region were included.

Identical genetic variants did not necessarily lead to a similar time of intervention nor life expectancy, which was previously shown by Faivre et al. [4]. They reported five *FBN1* variants leading to eoMFS that were also found in classical MFS. Remarkably, one of these variants—c.3143T>C (p.(Ile1048Thr)) in exon 25 leading to a new threonine predicted to give O‐glycosylation—was found in four individuals in our study. Two deceased at 3 and 24 months of age, while no AV‐valves surgery was performed. The other two underwent MV and TV annuloplasty surgery, and were 13 and 14 months of age at the last follow‐up. In a large genome‐wide association study, including 1070 pathogenic *FBN1* variants (although no variants in the neonatal region); nine modifier loci were found that were associated with aortic disease severity [39]. In another study, a maternally *inherited* splicing mutation, leading to skipping of exon 49, was found in an individual with an eoMFS phenotype whose affected mother had survived into adulthood [40], suggesting that modifiers were at play. Modifier studies have hardly been performed so far.

A potential limitation of our study was that we only included individuals with variants within the neonatal region (exon 24–32) of the *FBN1* gene, while Zarate et al. [12] reported that 4% of the individuals (3/77) who fulfilled the criteria for eoMFS were found to have an *FBN1* variant outside of the neonatal region, namely in exon 4, 33 and 35 [12, 41, 42]. Moreover, the UMD‐FBN1 mutation database reports 8 *FBN1* variants that cause eoMFS outside the neonatal region, namely in exon 4, 18–19, 20, 33, 33, 44–45, 53, and 57 (UMD_id: 437, 2245, 2222, 670, 308, 2307, 1307, and 2602 respectively; Request ID: 170123181251–337) [5, 43, 44, 45, 46]. Whether all these genetic variants would qualify for a diagnosis of eoMFS when using the novel scoring system [12] remains to be examined.

An additional limitation could be that we used the cut‐off point of 16 months of survival, based on the largest eoMFS review [7], whereas our data shows that the median age of death was 1 month which could instead have been used for our analyses. Yet, we think this would not change our main findings. A further limitation is that the improvement in medical care over the past decades could have been a confounder. For example, in 2006 ARBs were shown to decrease AoR growth in mouse models with MFS [47], and similar results were shown in humans [48]. Sixty‐two percent (15/24) of the individuals in our report deceased before 16 months were born before the introduction of ARBs in MFS patients around 2008. Therefore, new studies involving larger groups of children born in more recent years are needed to confirm our results.

## 5 Conclusions

EoMFS is a rare disorder with AVI being the most severe symptom, progressing in the first months of life. Similar to classical MFS, we propose to regard eoMFS as a spectrum, ranging from a severe disorder that is life‐threatening already before or immediately after birth, to a disorder with a better survival rate than previously thought, creating a window for AV‐valve surgery. Individuals with single amino acid substitutions had better survival compared to those with variants significantly impacting exon 24–32 length. Children who do survive past the first months of life, experience a variety of health issues requiring frequent follow‐up within a multidisciplinary team.

## Author Contributions

Conception and design: E.C.L., A.E.H., V.W., L.A.M.; Methodology: E.C.L., A.E.H., G.P., V.W., L.A.M.; Acquisition of data: E.C.L., A.E.H., G.P., L.Z., L.L., C.J., L.M., M.H., P.J., B.L.L.; Analysis: E.C.L.; Writing original‐draft: E.C.L.; Writing, review, and editing: V.W., L.A.M., A.E.H., G.P., L.L., C.J., L.M., M.H., P.J., B.L.L., J.W.‐K.; Supervision: V.W., L.A.M.

## Ethics Statement

The Medical Ethics Review Committee of the Amsterdam UMC delivered a waiver for our study (reference number W21_310 # 21.345). Of the nine enrolled individuals, parents (and children older than 12 years) provided written informed consent for the use and publication of the data.

## Conflicts of Interest

The authors declare no conflicts of interest.

## Peer Review

The peer review history for this article is available at https://www.webofscience.com/api/gateway/wos/peer‐review/10.1111/cge.14722.

## Supporting information


**Data S1.** Supporting Information.

**Supplemental Table S5.** Baseline summary of all individuals.

**Supplemental Table S6.** Overview genetic variants in FBN1 gene based on transcript NM_000138.4.

## Data Availability

The data that support the findings of this study are available on request from the corresponding author. The data are not publicly available due to privacy or ethical restrictions. The anonymized data of the individuals included in this study are available upon request.
