# Supplementary material for: Genotype–Phenotype Correlations, Treatment, and Prognosis of Children With Early‐Onset (Neonatal) Marfan Syndrome
Source: Clin Genet. 2025 Mar 10;108(2):134–45. doi: 10.1111/cge.14722 (PMC12215293; doi:10.1111/cge.14722)
Supplement: Supplementary file 1 — Data S1. Supporting Information. [file CGE-108-134-s003.docx]

**Supplemental Table S1-S4, and Supplemental Figure S1-S3.**

**Enrolled individuals**

| **Table S1** Scoring parameters of newly enrolled individuals based on eoMFS scoring scale^11^ | | | | | | | | | | | |  |
| --- | --- | --- | --- | --- | --- | --- | --- | --- | --- | --- | --- | --- |
|  |  | | | Individuals | | | | | | | |  |
| **Variable** | **Range** | 001 | 002 | | 005 | 006 | 010 | 011 | 013 | 014 | 024 | |
| **Cardiac score** |  |  |  | |  |  |  |  |  |  |  | |
| Mitral regurgitation | 0-5 | 5 | 3 | | 4 | 5 | 0-3^d^ | 3 | 0 | 5 | 4-5^h^ | |
| Mitral valve prolapse | 0-3 | 3 | 0-3^a^ | | 3 | 3 | 0-3^e^ | 3 | 3 | 3 | 3 | |
| Tricuspid regurgitation | 0-3 | 3 | 2 | | 0-2^b^ | 2 | 2 | 0-3^f^ | 3 | 3 | 3^i^ | |
| Aortic dilation | 0-4 | 3 | 3 | | 3 | 4 | 4 | 4 | N/A | 3 | N/A | |
| **Systemic score** |  |  |  | |  |  |  |  |  |  |  | |
| Arachnodactyly | 0-3 | 3 | 3 | | 3 | 3 | 3 | 3 | 3 | 3 | 3 | |
| Joint contractures | 0-3 | 3 | 0 | | 0 | 0 | 3 | 3 | 3 | 3 | 3 | |
| Pulmonary disease | 0-4 | 4 | 0 | | 0 | 0 | 0 | 0-4^g^ | 4 | 0 | 0 | |
| Lens dislocation | 0-4 | 0 | 4 | | 0 | 0 | 4 | 4 | 0 | 0 | 0 | |
| Facial features | 0-4 | 4 | 4 | | 0-4^c^ | 4 | 4 | 4 | 4 | 0 | 4 | |
| **FBN1 score** | 0-5 | 5 | 5 | | 5 | 5 | 5 | 5 | 5 | 5 | 5 | |
| Total score | 38 | 33 | 24-29 | | 18-24 | 26 | 25-31 | 24-31 | 25 | 25 | 25-26 | |
| **Note** Scoring based on eoMFS scoring scale of Zarate et al.^11^ The criteria of all different variables and their score ranges can be found in the supplementary files of their paper. In some cases it was unclear what the exact score was, therefore a range of probable scores and an explanation of the source of uncertainty was provided in these cases. **Abbreviations** eoMFS: early-onset Marfan syndrome **Superscripts** *a: Degree of prolapse is unknown, and no severe regurgitation is present; b: Grade 1.5; c: “facial dysmorphy” not further specified; d. Grade 1 regurgitation, annuloplasty at age 4 years (55 months); e: Prolapse at 9 months, no grade known; f: severe regurgitation developed after annuloplasty; g: Pneumothorax after removing drains of cardiac surgery; h: Detroit Z-score at age 2 years was 6.07; i: regurgitation present shortly after birth, grade unknown. Passed away at 5 weeks of cardiac/respiratory failure presumably caused by a pneumonia.* | | | | | | | | | | | |  |

| **Table S2** Textual medical summary of enrolled individuals | |
| --- | --- |
| **Individual** | **Textual summary** |
| 01 | This boy was diagnosed with eoMFS in his first month of life with mild MVI, and a dilated AoR (van Kessel et al. 2021) for which atenolol (2mg/kg) was prescribed. At 3 months of age he presented with tachypnea, TVI grade 1, MVI grade 2, and an AoR Z-score of 4.5, upon which losartan (0.7mg/kg) was added. At 9 months of age, he showed fatigue and excessive sweating. TVI grade 3, MVI grade 4, and AoR Z-score of 5.3 were noted. A NT-proBNP level of 767 pmol/l confirmed heart failure. Diuretics were started and an MV annuloplasty with 24mm Physio ring, and TV annuloplasty with 24 mm Physio ring took place. Because of a concomitant diaphragmatic hernia, a diaphragm plication was performed. After surgery tachy/dyspnea persisted and digoxin was prescribed for one month as inotropic agent to support the left ventricle. After three months the diuretics could be stopped. The last ultrasound (at 3.9 years of age) showed TVI grade 1, MVI grade 2, and an AoR Z-score of 5.9. He is currently 4 years of age and was diagnosed with autism spectrum disorder and intellectual disability of yet unclear origin. A gastrostomy was recently placed because of nutritional problems. |
| 02 | This boy was diagnosed at age 4 months with eoMFS, cardiac ultrasound showed AVI grade 1. Losartan (1.4 mg/kg) and atenolol (1.7 mg/kg) were prescribed at age 3 months and at age 6 months, respectively. When the child was almost 4 years old cardiac ultrasound showed prolapse of both AV-valves, AVI grade 2, and dilatation of the AoR (Z-score 5.8). No cardiac surgery has been performed. This boy is now almost 5 years old. |
| 05 | This male infant was diagnosed with eoMFS at age 10 months, cardiac ultrasound showed MVI grade 2, TVI grade 1, and a dilated AoR (Z-score Annulus 6.3, Sinus of Valsalva 5.0, ST-junction 4.0). Accordingly, atenolol (2mg/kg) and losartan (1.5mg/kg) were prescribed shortly thereafter. No cardiac surgery has been performed. At last follow up, at age 5.5 years, TVI was slightly increased, and the diameter of the AoR was increased, but Z-scores were overall stable or decreased (Annulus 6.5, Sinus of Valsalva 4.8, ST-junction 3.2). |
| 06 | First cardiac ultrasound at age 2 months of this male infant showed grade 3 insufficiency of both AV-valves, and a severe dilated AoR (Z-score 6.6). Atenolol was prescribed, and 2 months later propranolol was added. At 13 months cardiac ultrasound showed similar grade of AVI, a further dilated sinus of Valsalva (Z-score 7.2). At 15 months this infant got a VSRR (24mm graft), MV reconstruction with neochordea + annuloplasty and TV annuloplasty with plication of anteroseptal commissure. Last ultrasound at age 5.5 years showed MVI grade 2, and TVI grade 3. This boy is now almost 6 years old. |
| 10 | This boy was diagnosed at age 7 months, and cardiac ultrasound showed MVI grade 1, and a Z-score of the sinus of Valsalva of 4.1. Shortly thereafter atenolol was prescribed. At age 9 months TVI developed (grade 2). When he was 2 years old cardiac ultrasound showed an AoR Annulus Z-score of 3.1 and sinus of Valsalva Z-score of 4.6. At 4.6 years of age, AVI was not further increased, and the AoR Z score was 4.4, upon which cardiac surgery was performed (VSRR, MV annuloplasty (using 26mm Physio ring), and TV annuloplasty (using MC3-ring)). This boy is now almost 18 years old and doing remarkably well, although he experiences physical disabilities caused by several problems including severe scoliosis (Cobb angle of 56° at 6 years). |
| 11 | At age 1 month this boy was diagnosed with eoMFS, cardiac ultrasound showed TVI grade 1, and a dilated AoR (Z-score 4.4). Therefore, at 2 months of age atenolol (2mg/kg) was prescribed. Around 3 months Z-score of the AoR increased to 5.5, soon thereafter losartan (1mg/kg) was added. Around age 10 months the child developed deoxygenations consistently while sleeping, therefore oxygen was prescribed. Cardiac ultrasound at age 10 months showed no change in TVI, MVI grade 1, and a severe increase in Z-scores of the AoR (annulus 5.5, sinus of Valsalva 7.0, ST-junction 2.1) upon which VSRR (valsalva 24mm, and tube 18mm), and MV commisuroplasty and annuloplasty was performed. A few weeks after he presented with sweating. TVI grade 4 and a shortening fraction of 19% were noted, upon which furosemide and spironolactone were started. At age 16 months, the symptoms increased; he presented with tachypnea, excessive sweating, vomiting, and an enlarged liver. A second cardiac surgery was performed: TV annuloplasty with ring and plication of the right atrium. However, after surgery symptoms of heart failure persisted (NT-proBNP 10880 pmol/l) and high dosages of diuretics did not improve his symptoms. Digoxin was started because of supraventricular tachycardia. Aortic valve insufficiency was noted, MVI deteriorated, and NT-proBNP increased to 25000 pmol/l. After thorough discussions within the multidisciplinary team and with the parents, a third cardiac surgery (aortic valve, and MV surgery) was believed not to be in the boy’s best interest. Around age 19 months palliative care approach was chosen and the highest priority became the boy’s comfort. Therefore diuretics and fluid restriction were discontinued. A few days later palliative sedation was administered. |
| 13 | This girl showed dysmorphic features right after birth, therefore she was diagnosed shortly after birth with eoMFS (Bresters et al. 1999). Cardiac ultrasound showed significant prolapse of both AV-valves and dilation of the AoR. Pyloromyotomy was performed in the first few weeks of life for hypertrophic pyloric stenosis. Digoxin was prescribed because of paroxysmal supraventricular tachycardia (PSVT) with frequencies up to 260/min. At two months of age, she presented with tachypnea and excessive sweating. Cardiac ultrasound showed MVI grade 2-3, TVI grade 1-2, and aortic valve insufficiency grade 0-1 upon which propranolol (2mg/kg) and diuretics were prescribed. Digoxin was stopped. At 3 months she was admitted to the hospital for diagnosis of unexplained cyanotic events. During such an event in the hospital she had a cardiorespiratory arrest - with a sustained asystole on electrocardiographic monitoring - with quick recovery after resuscitation. A pacemaker was implanted and diaphragmatic eventration was repaired. After implantation sinus rhythm disappeared, and the pacemaker was activated (rate 70/min), but cardiac output stayed low. Even after providing supporting drugs, cardiac output was low and considering the prognosis, the regime was changed into palliative care. She died at age 3 months of dysrhythmia and severe MVI – confirmed on ultrasound shortly before death. In the autopsy report abnormal tissue surrounding the AV-node was noticed. |
| 14 | This boy passed away directly after birth due to cardiac- and respiratory failure. A few of his morphological features are shown in Figure S1. Other features were smooth, and flat ears, and contractures of hips and knees. The autopsy report described an extremely dilated heart, intrathoracic fluid, and ascites. Dilated atria with thin walls, a small right ventricle with also thin walls, and a dilated AoR. |
| 24 | Of this boy no specific cardiac parameters were available (van Putte-Katier and Hofkamp 2007). However, it is known that severe MVI and TVI was present at birth. This boy passed away at age one month, because of severe cardiorespiratory failure, and dyspnea caused by a respiratory infection. |
| ***Abbreviations*** *eoMFS: Early-Onset Marfan syndrome; MVI: Mitral Valve Insufficiency; AoR: Aortic Root; TVI: Tricuspid Valve Insufficiency; MV: Mitral Valve; TV: Tricuspid Valve; AVI: Atrioventricular Valve Insufficiency;* AV: Atrioventricular; *VSRR: V*alve Sparing aortic Root Replacement | |


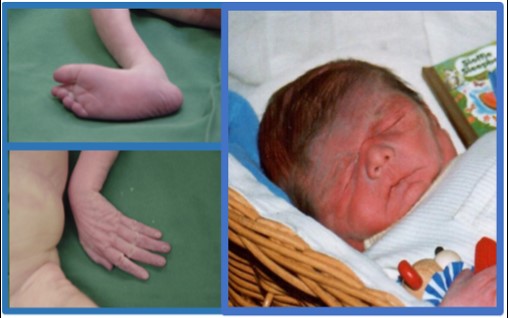


**Figure S1**. Morphological features of a newborn with early onset Marfan syndrome. Pictures taken from male infant (individual 14). Please note the loose facial skin, retrognathia, long fingers and long foot.

**Individuals included from literature**


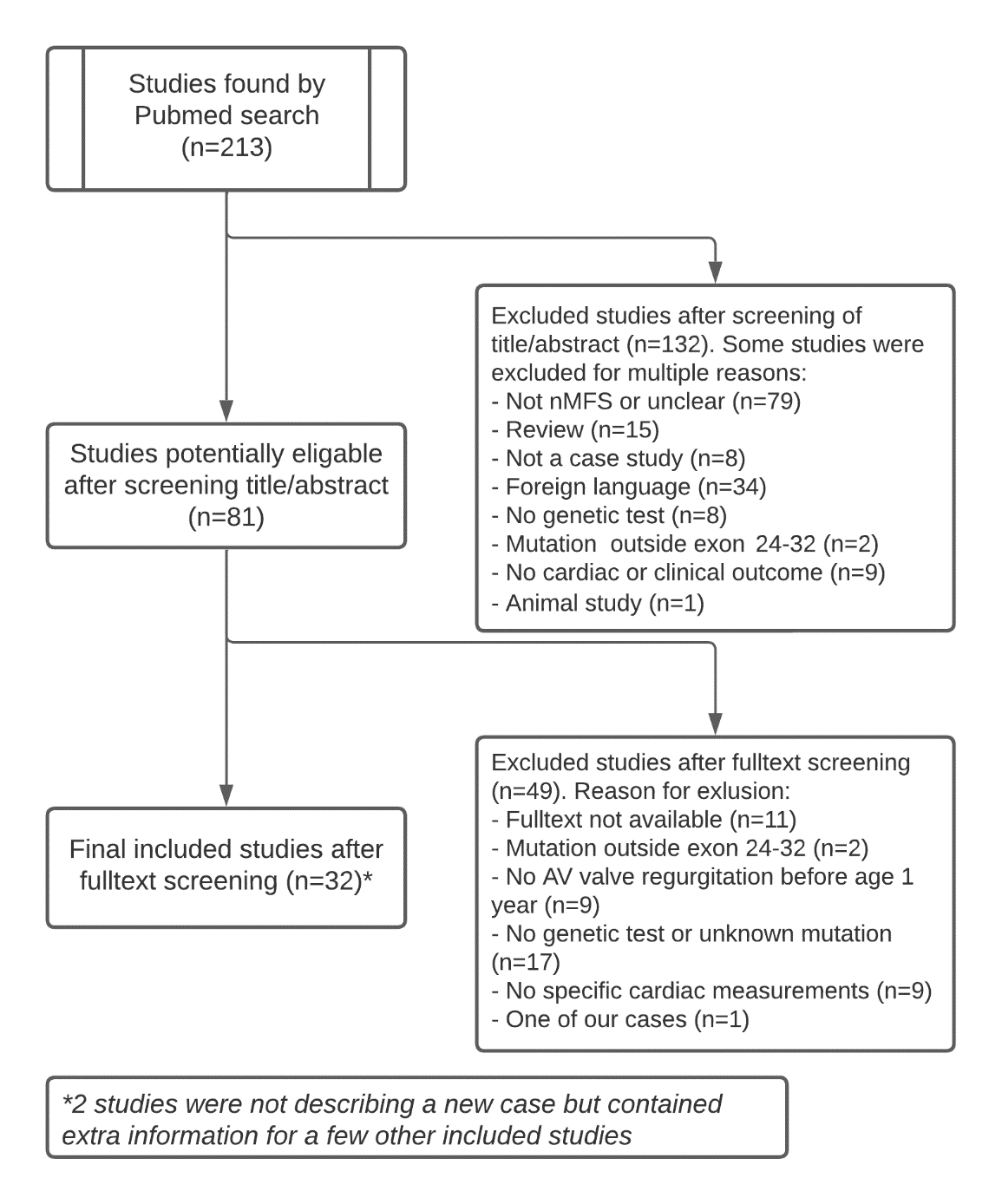


**Figure S2**. Flow-chart describing method for inclusions from literature.
**Note** *Two studies were included for the extra information they contained regarding already included individuals and did not contain any new cases for this study (Godfrey et al. 1995, Kainulainen et al. 1994). Two studies contained information for multiple individuals who met the inclusion criteria (Ardhanari et al. 2019: two individuals; Gavilan et al. 2011: two individuals).

| Table S3 References for individuals additionally included from literature search | | |
| --- | --- | --- |
| ID | Study | ID in original study |
| L01 | (Amado et al. 2014) | Individual 1/1 |
| L02 | (Apitz et al. 2010) | Individual 1/1 |
| L03 | (Ardhanari, Barbouth, and Swaminathan 2019) | Patient 1/3 |
| L04 | (Ardhanari, Barbouth, and Swaminathan 2019) | Patient 2/3 |
| L05 | (Barnett et al. 2010) | Patient 2/2 |
| L06 | (Booms et al. 1999; Godfrey et al. 1995) | Individual 1/1; ID 775 |
| L07 | (Buntinx et al. 1991; Kainulainen et al. 1994) | Individual 1/1; K125N mutation |
| L08 | (Carande, Bilton, and Adwani 2017) | Individual 1/1 |
| L09 | (Chao et al. 2010) | Individual 1/1 |
| L10 | (Cua et al. 2020) | Individual 1/1 |
| L11 | (Derbent et al. 2008) | Individual 1/1 |
| L12 | (Gavilan et al. 2011) | Case 1/2 |
| L13 | (Gavilan et al. 2011) | Case 2/2 |
| L14 | (Hanséus et al. 1995; Kainulainen et al. 1994) | Individual 1/1; DEL1EGF mutation |
| L15 | (ter Heide et al. 2005) | Individual 1/1 |
| L16 | (Kawamura, Ueno, and Kawano 2021) | Individual 1/1 |
| L17 | (Liu et al. 2018) | Individual 1/1 |
| L18 | (Lopes et al. 2006) | Individual 1/1 |
| L19 | (Ng et al. 1999) | Individual 1/1 |
| L20 | (Peng et al. 2016) | Individual 1/1 |
| L21 | (Raghunath et al. 1993; Kainulainen et al. 1994) | Individual 1/1; C176R mutation |
| L22 | (Revencu et al. 2004) | Individual 1/1 |
| L23 | (Shinawi et al. 2005) | Individual 1/1 |
| L24 | (Solé-Ribalta et al. 2019) | Individual 1/1 |
| L25 | (Sutherell et al. 2007) | Individual 1/1 |
| L26 | (Tekin et al. 2007) | Patient 1/3 |
| L27 | (Tognato et al. 2019) | Individual 1/1 |
| L28 | (Wang et al. 1997) | Individual 1/1 |
| L29 | (Weidenbach et al. 1999) | Individual 1/1 |
| L30 | (Whitelaw et al. 2004) | Patient 1/2 |
| L31 | (Wojcik et al. 2019) | Individual 1/1 |
| L32 | (Yoon and Kong 2021) | Individual 1/1 |

**All individuals**

**
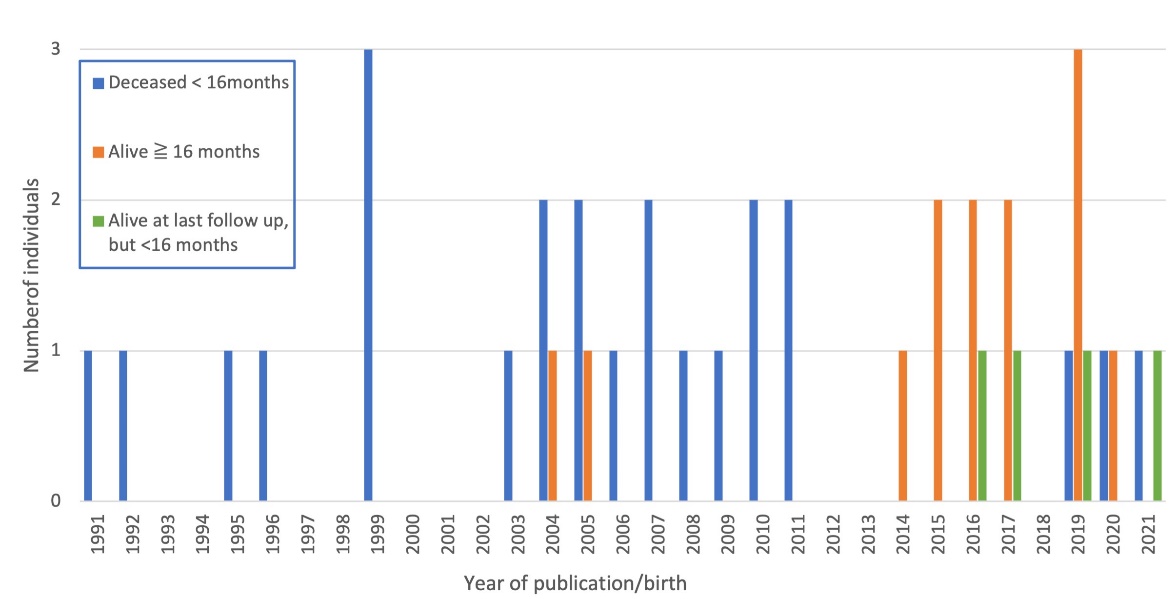
**

**Figure S3** Group survival over time. Overview of number of individuals per group born in a certain year. **Note** For previously published individuals year of birth was unknown, therefore publication year was used.

| Table S4 Recurrent genetic variants |
| --- |
| We found five recurrent variants. Two of these recurrent variants were found in individuals distributed over the two groups A and B. The first of these variants, c.3143T>C (p.(Ile1048Thr) in exon 25 was found in four individuals (13, L17, L08, L27). Two of these individuals deceased, at 3 months (individual 13, born before 2000) and at 24 months of age (individual L17). In neither of them, AV-valve surgery had been performed. The other two underwent MV and TV surgery and were 13 and 14 months of age at last follow-up (Figure 2B). L17, L08, L27 were patients from studies published between 2016-2019 (Table S5). The second variant, the canonical splice-acceptor-site variant c.3965-2A>G in intron 31, which is predicted to lead to skipping of exon 32, was found in individual 11 and L23 (born in 2015, and at least a decade before, respectively). Both individuals were deceased; the first at the age of 19 months and the other at the age of 4 months. Individual 11 underwent VSRR and MV annuloplasty at age 12 months, and TV annuloplasty with ring at age 16 months. L23 had a balloon dilatation of pulmonary valve at one month.  Four other individuals (24, L06, L13, L32) had the genetic variant c.3964+1G>T (in intron 31), suspected to lead to a deletion of the 17^th^ EGF-like domain. All these individuals were deceased; three at one month of age and the fourth at four months of age (individual L06). These children were all born in a different decade, namely around 2005, 1999, 2011 and 2021, respectively (Table S5). |
